# Elevated EZH2 expression is associated with reduced NRF2-related antioxidant signaling and ferroptosis-associated features in eosinophilic chronic rhinosinusitis with nasal polyps

**DOI:** 10.3389/fmed.2026.1966049

**Published:** 2026-10-28

**Authors:** Guan-Jiang Huang, Xin-Ru Liu, Biao-Qing Lu, Hong-Bing Liu

**Affiliations:** 1 Department of Otorhinolaryngology Head and Neck Surgery, Zhongshan Hospital of Traditional Chinese Medicine, Affiliated to Guangzhou University of Chinese Medicine, Zhongshan, Guangdong, China; 2 The Tenth Clinical Medical College of Guangzhou University of Chinese Medicine, Zhongshan, Guangdong, China; 3 Department of Otolaryngology, Head and Neck Surgery, The Second Affiliated Hospital, Jiangxi Medical College, Nanchang University, Nanchang, Jiangxi, China

**Keywords:** eosinophilic chronic rhinosinusitis with nasal polyps, epigenetic regulation, EZH2, ferroptosis, GPX4, NRF2, oxidative stress, type 2 inflammation

## Abstract

Recurrence remains common in eosinophilic chronic rhinosinusitis with nasal polyps (eCRSwNP) despite surgery and type 2-directed therapy, suggesting that epithelial injury mechanisms beyond cytokine signaling remain incompletely defined. Epigenetic-redox dysregulation is a plausible contributor, but its relationship to NRF2-associated antioxidant defenses in eCRSwNP has received little attention. Current therapies suppress type 2 inflammation but may not directly address persistent epithelial redox imbalance, providing a rationale for examining complementary regulatory pathways in patients with severe recurrent disease. We explored associations among EZH2 expression, NRF2-related antioxidant readouts, oxidative injury, and ferroptosis-associated features using patient sinonasal tissues, primary nasal epithelial cells subjected to EZH2-targeting shRNA, and an exploratory AAV-treated mouse model. In human tissues, higher EZH2 expression accompanied lower NRF2-related antioxidant marker levels and a ferroptosis-associated expression pattern. In cultured cells, EZH2 targeting was accompanied by improved antioxidant readouts, lower intracellular oxidation, and less mitochondrial injury. The mouse experiment showed parallel treatment-associated differences in protein staining and sinonasal histopathology, although EZH2 target engagement in nasal mucosa was not confirmed. These findings do not establish ferroptosis because functional rescue, iron-dependence testing, and direct phospholipid-peroxidation measurements were not performed. They also do not establish NRF2 dependence, direct EZH2–NRF2 binding, or the temporal direction of the relationship among EZH2, NRF2-related readouts, and oxidative stress. The results therefore identify a preliminary epigenetic-redox association rather than a defined EZH2–NRF2 mechanism. This framework may help guide future studies incorporating time-resolved measurements, reciprocal genetic perturbation, NRF2 localization and activity assays, ferroptosis-selective rescue, and direct validation of *in vivo* target engagement.

## 1 Introduction

Chronic rhinosinusitis is a heterogeneous inflammatory condition of the nasal and paranasal mucosa that affects a substantial proportion of adults worldwide and imposes a considerable burden on quality of life and healthcare systems (1). Global systematic reviews estimate that chronic rhinosinusitis affects between 5 and 28 percent of adults, with chronic rhinosinusitis with nasal polyps representing a clinically severe subgroup (1–3). Within this population, eosinophilic chronic rhinosinusitis with nasal polyps (eCRSwNP) is recognized as the endotype that most consistently reflects an underlying type 2 immune process (4, 5). Comparative transcriptomic and endotyping studies have further delineated the molecular heterogeneity of CRSwNP by distinguishing aspirin-exacerbated respiratory disease (AERD) from non-AERD subgroups through distinct immune gene expression signatures (6). According to widely used histopathological criteria, tissue eosinophilia exceeding ten eosinophils per high-power field defines the eosinophilic subtype, which is characterized by dense eosinophilic infiltration, elevated interleukin 4, interleukin 5, and interleukin 13 levels, and a markedly higher postoperative recurrence rate than the non-eosinophilic subtype (7–9).

The pathogenesis of eCRSwNP is now understood to involve a self-amplifying circuit in which damaged epithelium releases alarmins that activate group 2 innate lymphoid cells and T helper 2 cells; these cells, in turn, secrete interleukin 4, interleukin 5, and interleukin 13 to sustain eosinophil recruitment, goblet cell hyperplasia, and tissue remodeling (10–12). Comparative transcriptomic studies have shown that eosinophilic and non-eosinophilic nasal polyps have distinct inflammatory signatures, suggesting endotype-specific molecular drivers rather than a single unifying mechanism (13). Biologic agents targeting interleukin 4 receptor alpha, interleukin 5, and immunoglobulin E have transformed the management of severe disease, yet a meaningful proportion of patients remain refractory or experience recurrence after both surgery and biologic therapy (14, 15). Emerging evidence from the epithelial barrier framework further indicates that oxidative stress and barrier injury are early, central events that precede and amplify type 2 inflammation in the airway mucosa, positioning redox regulation and epigenetic control as compelling areas for mechanistic investigation in eCRSwNP (16, 17).

Ferroptosis is a form of regulated cell death first defined by Dixon and colleagues and is characterized by the iron-dependent accumulation of lipid peroxides. It is morphologically distinguished from apoptosis by shrunken mitochondria with condensed membranes and diminished cristae (18–20). Subsequent work has delineated an integrated molecular system in which glutathione peroxidase 4 restrains phospholipid peroxidation, the cystine-glutamate antiporter system Xc- maintains glutathione synthesis, and acyl-coenzyme A synthetase long-chain family member 4 promotes the incorporation of polyunsaturated fatty acids into membrane phospholipids, thereby sensitizing cells to peroxidation (21–23). Ultrastructural studies consistently identify shrunken, electron-dense mitochondria with reduced or absent cristae as a defining hallmark that can be visualized by transmission electron microscopy (24–26). In the airway, ferroptosis has been linked to eosinophil biology. Ferroptosis-inducing agents trigger a noncanonical, lipid peroxidation-independent form of eosinophil death that alleviates allergic airway inflammation, whereas cold exposure and interleukin 13-driven suppression of SLC7A11 promote epithelial ferroptosis and exacerbate allergic airway disease (27–31). In chronic rhinosinusitis with nasal polyps specifically, bioinformatic analyses using the FerrDb and Gene Expression Omnibus resources have identified differentially expressed ferroptosis-related genes and proposed several hub genes with diagnostic potential (32–35). A recent report identified the RNA-binding protein ZFP36L1 as a driver of ferroptosis through destabilization of CAMK2A messenger RNA and impairment of mitochondrial quality control (34). Separately, glutathione peroxidase 4-regulated neutrophil ferroptosis has been shown to trigger systemic autoimmunity. These studies, including our previous work, support a pathogenic role for ferroptosis in chronic rhinosinusitis with nasal polyps; however, the upstream regulatory circuitry controlling this process in the eosinophilic endotype has not been mechanistically defined.

Nuclear factor erythroid 2-related factor 2, commonly known as NRF2, is a master transcriptional regulator of the cellular antioxidant response and a central determinant of ferroptosis susceptibility (36). Under basal conditions, NRF2 is bound by KEAP1 and targeted for proteasomal degradation. In contrast, oxidative stimuli release NRF2, allowing it to translocate to the nucleus and induce antioxidant response element-driven genes, including heme oxygenase 1 and glutathione peroxidase 4, thereby restraining lipid peroxidation and ferroptotic cell death (36–39). Given this protective function, loss of NRF2 activity has been implicated in ferroptosis-associated injury across a range of tissues, and pharmacological or genetic restoration of NRF2 signaling consistently reduces ferroptotic markers and tissue damage (40, 41). Whether NRF2 activity is suppressed in eCRSwNP and the upstream mechanism responsible have not previously been examined.

EZH2 is the catalytic subunit of polycomb repressive complex 2 and mediates trimethylation of histone H3 at lysine 27 to silence target gene transcription. It also exerts polycomb repressive complex 2-independent, noncanonical functions through direct protein interactions (42–44). Beyond its extensively characterized oncogenic role, EZH2 restrains T helper 2 differentiation. Loss of EZH2 in CD4-positive T cells promotes exaggerated allergic airway inflammation and increased production of interleukin 4 and interleukin 5, and human data indicate that EZH2 expression in T helper cells is inversely associated with allergic rhinitis (45–47). EZH2 has also been implicated in a growing number of nonmalignant inflammatory and fibrotic conditions, including inflammatory bowel disease, acute respiratory distress syndrome-associated pulmonary fibrosis, and renal fibrosis, in which pharmacological EZH2 inhibition consistently attenuates tissue injury (48–50). Notably, EZH2-mediated hypomethylation has been reported to epigenetically silence the NFE2L2 promoter, which encodes NRF2, in colon cancer, raising the possibility of a direct regulatory link between EZH2 and NRF2 that has not been explored in airway mucosal disease (42, 51). Given that EZH2 is an established negative regulator of type 2 immune restraint and a candidate upstream repressor of NRF2, we hypothesized that EZH2 is upregulated in eCRSwNP and affects ferroptosis-associated epithelial injury through suppression of NRF2 signaling.

We investigated whether increased EZH2 expression co-occurs with reduced NRF2-related antioxidant signaling, oxidative stress, and ferroptosis-associated features in eCRSwNP. The study was designed to identify a candidate molecular association and generate testable hypotheses. It was not designed to determine whether changes in EZH2 precede oxidative stress, whether reactive oxygen species (ROS) induce EZH2 expression, or whether the observed changes constitute a bidirectional feedback relationship.

## 2 Materials and methods

### 2.1 Clinical samples and ethical approval

Eighteen nasal mucosal specimens were obtained from patients undergoing endoscopic sinus surgery or inferior turbinate reduction. The control group (*n* = 6) comprised normal inferior turbinate mucosa from patients undergoing partial inferior turbinectomy for structural nasal obstruction. Nasal polyp tissue (*n* = 12) was collected from patients with chronic rhinosinusitis with nasal polyps who underwent functional endoscopic sinus surgery. All available hematoxylin and eosin-stained nasal polyp sections were independently reevaluated by two experienced pathologists who were blinded to the molecular results, previous group allocation, and each other's counts. Each section was first examined at low magnification to identify representative viable polyp tissue. Eosinophils were then counted in five nonoverlapping high-power fields at 400× magnification using the same microscope settings for all specimens. The mean eosinophil count across the five fields was calculated for each specimen. Samples with a mean count exceeding 10 eosinophils per high-power field were classified as eosinophilic nasal polyps, whereas those with a mean count of 10 or fewer eosinophils per high-power field were classified as non-eosinophilic nasal polyps. Cases for which the two assessments resulted in discordant classifications were jointly reviewed to reach consensus. The polyp samples were further divided into a non-eosinophilic nasal polyp group (NP, *n* = 6) and an eosinophilic nasal polyp group (eNP, *n* = 6). Each specimen was divided immediately after excision; one portion was fixed in four percent paraformaldehyde for histological analysis, and the remainder was stored at negative eighty degrees Celsius for molecular analysis. All procedures involving human tissue were performed with informed consent and institutional ethical approval.

### 2.2 Cell culture and grouping

Primary human nasal mucosal epithelial cells and primary human nasal polyp mucosal epithelial cells were obtained commercially and cultured in dedicated epithelial cell medium under standard conditions of 37 °C and 5% CO_2_. Cells in the logarithmic growth phase were used for all experiments. Four experimental groups were established: a control group, a model group representing untreated nasal polyp epithelial cells, a model plus Sh-EZH2 group, and a model plus Sh-EZH2 plus NRF2 agonist group. In the model plus Sh-EZH2 group, cells were transduced with an EZH2-targeting short hairpin RNA (shRNA) lentivirus at a multiplicity of infection (MOI) of 20, followed by puromycin selection at 1 μg/mL. In the model plus Sh-EZH2 plus NRF2 agonist group, the NRF2 agonist curcumin was additionally applied at 10 μM. A non-targeting lentivirus served as the negative control for the knockdown construct.

### 2.3 Animal model and grouping

Eighteen three-week-old male BALB/c mice were obtained from a licensed commercial supplier and housed under specific pathogen-free (SPF) conditions at 20 °C to 26 °C and 40% to 70% relative humidity, with free access to food and water. The animals were randomly allocated to three groups of six: a control group, an eCRSwNP model group, and a model plus AAV-m-EZH2-shRNA1 group. The eCRSwNP model was induced using an established ovalbumin and staphylococcal enterotoxin B protocol. Mice received intraperitoneal sensitization with ovalbumin adsorbed to aluminum hydroxide on days 1 and 5, followed by intranasal ovalbumin challenge from day 12 to day 19. This was followed by a maintenance phase involving intranasal ovalbumin three times weekly with concurrent weekly staphylococcal enterotoxin B instillation, a regimen consistent with previously validated murine models of eosinophilic nasal polyposis (52–54). After model establishment, the model group received intranasal blank adeno-associated virus, and the intervention group received intranasal AAV-m-EZH2-shRNA1, each at a titer of one times ten to the tenth plaque-forming units per nostril; the control group received an equivalent volume of saline throughout sensitization and challenge.

### 2.4 Co-immunoprecipitation

The association between EZH2 and NRF2 was assessed by co-immunoprecipitation. Nasal polyp epithelial cells were lysed in ice-cold lysis buffer containing protease and phosphatase inhibitors, briefly sonicated, and clarified by centrifugation. The lysates were precleared with protein A and protein G beads and incubated overnight at four degrees Celsius with an antibody against the target protein or an isotype-matched control immunoglobulin. Immune complexes were then captured with protein A and protein G beads, washed extensively, eluted in sodium dodecyl sulfate sample buffer, and analyzed by western blotting as described below.

### 2.5 Western blot analysis

Total protein was extracted from cultured cells or homogenized nasal tissue using radioimmunoprecipitation assay lysis buffer supplemented with protease and phosphatase inhibitors. Protein concentrations were determined using the bicinchoninic acid method. Equal amounts of protein were separated by sodium dodecyl sulfate–polyacrylamide gel electrophoresis using ten percent gels, transferred to polyvinylidene fluoride membranes, blocked with five percent nonfat milk, and incubated overnight at four degrees Celsius with primary antibodies against EZH2, NRF2, GPX4, HMOX1, PTGS2, and beta-actin; antibody details are provided in Table 1. The membranes were then incubated with a horseradish peroxidase-conjugated secondary antibody and visualized using enhanced chemiluminescence on an automated imaging system. Band intensities were normalized to beta-actin.

**Table 1:** Antibodies.

Antibodies	Manufacturer	Product number
*β*-ACTIN Rabbit RecAb	Proteintech	81115-1-RR
EZH2 Polyclonal antibody	Proteintech	21800-1-AP
NRF2,NFE2L2 Polyclonal antibody	Proteintech	16396-1-AP
Anti-GPX4 Antibody(Clone#ACCO-7)	BOSTER	BM5231
HO-1/HMOX1 Polyclonal antibody	Proteintech	10701-1-AP
COX2/PTGS2 Polyclonal antibody	Proteintech	12375-1-AP
HRP conjugated Goat Anti-Rabbit IgG (H + L)	Servicebio	GB23303

### 2.6 RNA extraction and quantitative real-time polymerase chain reaction

Total RNA was extracted from tissues and cells using a column-based extraction kit. RNA concentration and purity were verified by ultraviolet spectrophotometry, with 260/280 nm absorbance ratios of approximately 2.0 for all samples. Complementary DNA was synthesized after genomic DNA removal, and quantitative polymerase chain reaction was performed using SYBR Green-based chemistry on a real-time thermal cycler with a two-step amplification protocol. Relative gene expression was calculated using the 2^−*ΔΔ*Ct^ method, with glyceraldehyde three phosphate dehydrogenase as the internal reference. Primer sequences for EZH2, NRF2, HMOX1, GPX4, PTGS2, and glyceraldehyde three phosphate dehydrogenase are listed in Table 2.

**Table 2:** Primer sequences used for qRT-PCR (5’-3’).

Gene	Primer	Sequence 5’-3’
GAPDH	Forward Primer	ACACCCACTCCTCCACCTTTG
	Reverse Primer	TCCACCACCCTGTTGCTGTAG
GPX4	Forward Primer	CGCTGTGGAAGTGGATGAAGATC
	Reverse Primer	TGTCGATGAGGAACTGTGGAGAG
EZH2	Forward Primer	TGACAGTTCGTGCCCTTGTGTG
	Reverse Primer	ACTCTCGGACAGCCAGGTAGC
Nrf2	Forward Primer	TTCCTTCAGCAGCATCCTCTCC
	Reverse Primer	AATCTGTGTTGACTGTGGCATCTG
CAV1	Forward Primer	GCAGAACCAGAAGGGACACAC
	Reverse Primer	CAAAGAGGGCAGACAGCAAGC
IFNGR1	Forward Primer	GTGAAGAGCCGTTGTCTCCA
	Reverse Primer	ATTGGAGTCAGATGGCTGCC
HMOX1	Forward Primer	GCCAGTGCCACCAAGTTCAAG
	Reverse Primer	GATGTTGAGCAGGAACGCAGTC
PTGS2	Forward Primer	TCCACCAACTTACAATGCTGACTATG
	Reverse Primer	ATCATCAGGCACAGGAGGAAGG

### 2.7 Cell viability assay

Cell viability was assessed using a Cell Counting Kit-8 (CCK-8) assay. Cells were seeded at a density of 2.5 × 10^4^ cells per well in 96-well plates, allowed to adhere for twenty-four hours, and then treated according to group assignment for 0, 24, 48, or 72 h. CCK-8 reagent was added, and the plates were incubated for two hours before absorbance was measured at 450 nm using an automated microplate reader. Relative viability was calculated from the blank and treatment absorbance values.

### 2.8 Measurement of intracellular reactive oxygen species

Intracellular reactive oxygen species were measured using the 2’,7’-dichlorodihydrofluorescein diacetate (DCFH-DA) fluorescent probe. Cells seeded in black-walled 96-well plates were incubated with 10 μM of the probe for 30 min at 37 °C, washed with phosphate-buffered saline, and analyzed for fluorescence intensity at an excitation wavelength of four hundred eighty-five nanometers and an emission wavelength of five hundred twenty-five nanometers using a multimode microplate reader.

### 2.9 Transmission electron microscopy

Ferroptosis-associated mitochondrial ultrastructure was evaluated by transmission electron microscopy. Cells were fixed in electron microscopy fixative, gently detached, pelleted, refixed, and subsequently processed by a commercial electron microscopy service for ultrathin sectioning and imaging to assess mitochondrial size, membrane density, and cristae integrity across experimental groups.

### 2.10 Histology and immunohistochemistry

Paraffin-embedded tissue sections were deparaffinized, rehydrated through a graded ethanol series, and subjected to antigen retrieval using citrate buffer. Endogenous peroxidase activity was quenched with 3% hydrogen peroxide. Sections were incubated overnight at 4 °C with primary antibodies against GPX4, FTH1, SLC7A11, and ACSL4, followed by a species-appropriate secondary antibody using a two-step immunohistochemistry detection system. Sections were visualized with diaminobenzidine substrate and counterstained with hematoxylin. For general histopathological assessment, parallel sections were stained with hematoxylin and eosin. Slides were imaged by light microscopy and analyzed using Image-Pro Plus software to determine integrated optical density normalized to area as a semiquantitative measure of protein expression.

### 2.11 Statistical analysis

All data are expressed as mean ± standard deviation and were analyzed using GraphPad Prism version 9.0. Comparisons among three or more groups were performed using one-way analysis of variance followed by Dunnett *post hoc* testing when homogeneity of variance was confirmed. Comparisons between two groups were performed using an independent-samples t test. A P value < 0.05 was considered statistically significant; significance is denoted throughout as *P < 0.05, **P < 0.01, and ***P < 0.001.

## 3 Results

### 3.1 EZH2 is upregulated and ferroptosis-associated markers are altered in eCRSwNP clinical tissues

To characterize the expression pattern of EZH2 and ferroptosis-related molecules in eCRSwNP, tissue samples from the control, non-eosinophilic NP, and eNP groups were analyzed by western blotting and quantitative reverse-transcription polymerase chain reaction (Supplementary Figure S1). Both mRNA and protein levels of EZH2 were significantly higher in eNP tissue than in non-eosinophilic NP and control tissues. In parallel, NRF2 and its downstream antioxidant effectors, HMOX1 and GPX4, were markedly reduced in eNP tissue at both the protein and transcript levels. Among the additional ferroptosis-associated markers examined, PTGS2 showed the greatest upregulation. These findings indicate a coordinated expression pattern involving several proteins commonly assessed in ferroptosis research. However, none of these markers is specific to ferroptosis; their combined alteration should not be interpreted as direct evidence of ferroptotic cell death.

### 3.2 EZH2 and NRF2 are detected in the same immunoprecipitate in nasal polyp epithelial cells

NRF2 immunoreactivity was detected in EZH2-containing immunoprecipitates from primary nasal polyp epithelial cell lysates (Figure 1A). This finding indicates biochemical co-association under the experimental lysis and immunoprecipitation conditions but does not distinguish direct binary binding from indirect association within a multiprotein complex. Furthermore, the experiment does not determine whether this co-association affects NRF2 nuclear localization, transcriptional activity, protein stability, ubiquitination, or degradation.

**Figure 1:**
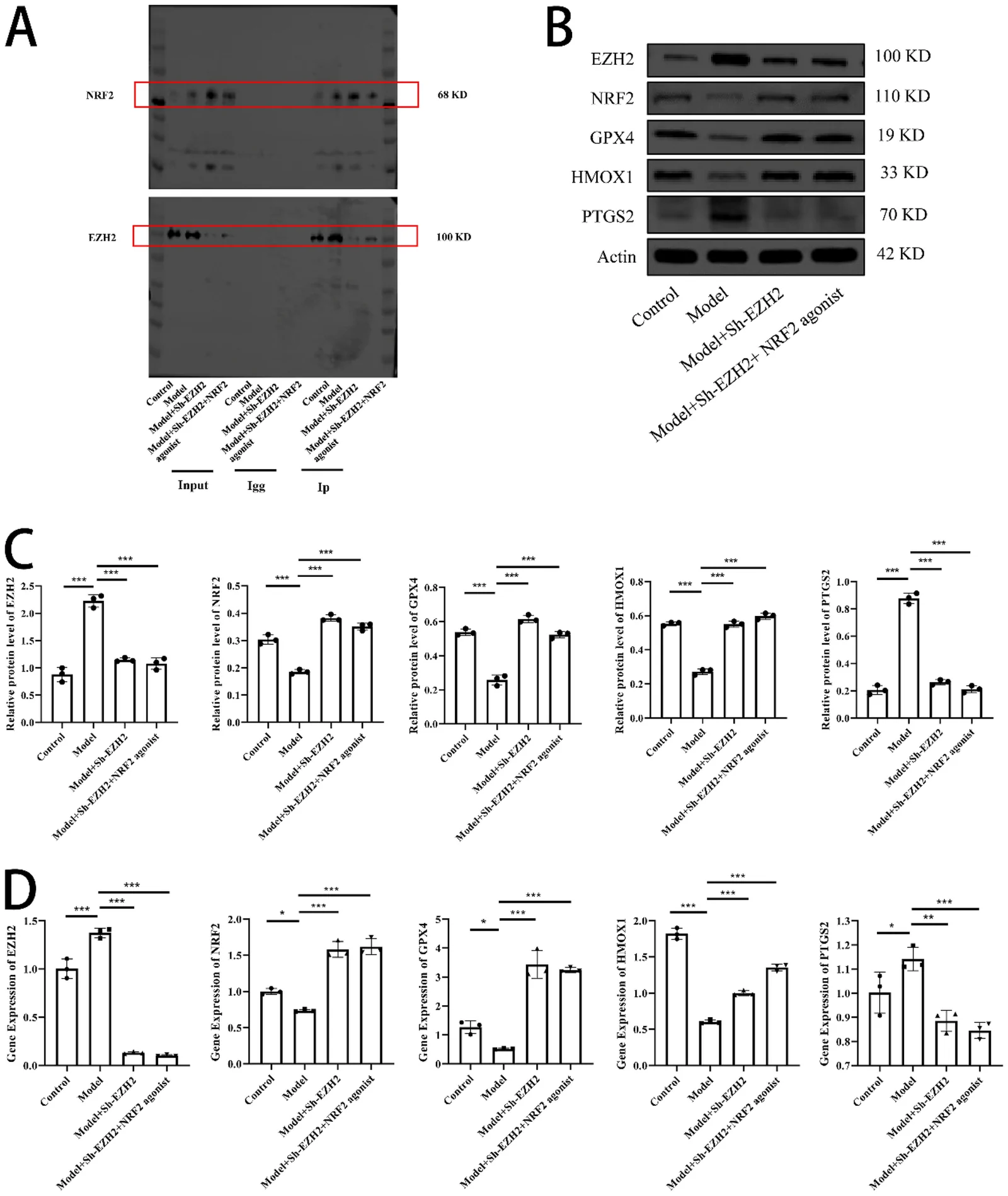
Endogenous EZH2 and NRF2 co-immunoprecipitate, and EZH2 knockdown is associated with changes in NRF2-related antioxidant signaling and ferroptosis-associated markers. **(A)** Co-immunoprecipitation of endogenous EZH2 and NRF2 in primary nasal polyp epithelial cells. For each experimental group, the lanes labeled “Input” contained an aliquot of the corresponding total cell lysate collected before immunoprecipitation. Immunoprecipitation was performed using 500 μg of total protein and 2 μg of anti-EZH2 antibody (Proteintech, 21800-1-AP). An equal amount of isotype-matched rabbit IgG was used in parallel as the negative immunoprecipitation control (“IgG”), and immune complexes were captured using Protein A/G magnetic beads. The resulting immunoprecipitates (“IP”) were immunoblotted with anti-NRF2/NFE2L2 antibody (Proteintech, 16396-1-AP); probing for EZH2 documented recovery of the immunoprecipitated protein. This result supports biochemical co-association but does not establish direct binary binding. **(B)** Representative western blot images and **(C)** densitometric quantification of EZH2, NRF2, GPX4, HMOX1, and PTGS2 protein expression in the control, model, model plus Sh-EZH2, and model plus Sh-EZH2 plus NRF2 agonist groups. **(D)** Quantitative reverse-transcription polymerase chain reaction analysis of the corresponding mRNA levels. Data are presented as mean ± standard deviation. *P < 0.05, **P < 0.01, and ***P < 0.001 for the indicated comparisons.

### 3.3 EZH2 knockdown is associated with NRF2 pathway activation and reversal of ferroptosis-associated marker changes

Western blotting and quantitative polymerase chain reaction analyses were performed across the control, model, model plus Sh-EZH2, and model plus Sh-EZH2 plus NRF2 agonist groups (Figures 1B-D). Relative to the control group, the model group exhibited significantly elevated EZH2 and PTGS2 expression together with markedly reduced GPX4 and HMOX1 at both the mRNA and protein levels. Following EZH2 knockdown, NRF2-associated antioxidant signaling increased, accompanied by reversal of several ferroptosis-associated marker changes. EZH2-targeting shRNA treatment was accompanied by increases in NRF2-related antioxidant markers and reductions in oxidative-stress-associated measurements. However, because these variables were assessed only at established endpoints, their temporal order cannot be determined. Furthermore, the absence of an additional effect after curcumin treatment does not establish NRF2 dependence, pathway epistasis, or a unidirectional EZH2-to-NRF2 relationship.

### 3.4 EZH2 knockdown attenuates oxidative stress and is accompanied by improved cell viability and mitochondrial morphology

Functional assessment showed that the model group had elevated inflammatory activity and substantially increased cellular oxidative stress (Figure 2). Transmission electron microscopy revealed disrupted mitochondrial architecture in the model group, with cristae fracture and pronounced ultrastructural disorganization described as characteristic of ferroptosis (Figure 2A). Cell Counting Kit-8 (CCK-8) assay results showed that EZH2 knockdown partially restored cell viability toward levels approaching those of the control group (Figure 2B). Both the model plus Sh-EZH2 group and the model plus Sh-EZH2 plus NRF2 agonist group exhibited significantly lower reactive oxygen species fluorescence intensity than the model group (Figures 2C,D). The model group exhibited increased DCFH-DA fluorescence and mitochondrial alterations, including cristae disruption and mitochondrial shrinkage. DCFH-DA reflects general intracellular oxidation rather than phospholipid peroxidation, and the observed mitochondrial abnormalities are not pathognomonic for ferroptosis. These results therefore demonstrate oxidative and mitochondrial injury but do not identify a ferroptotic mode of cell death.

**Figure 2:**
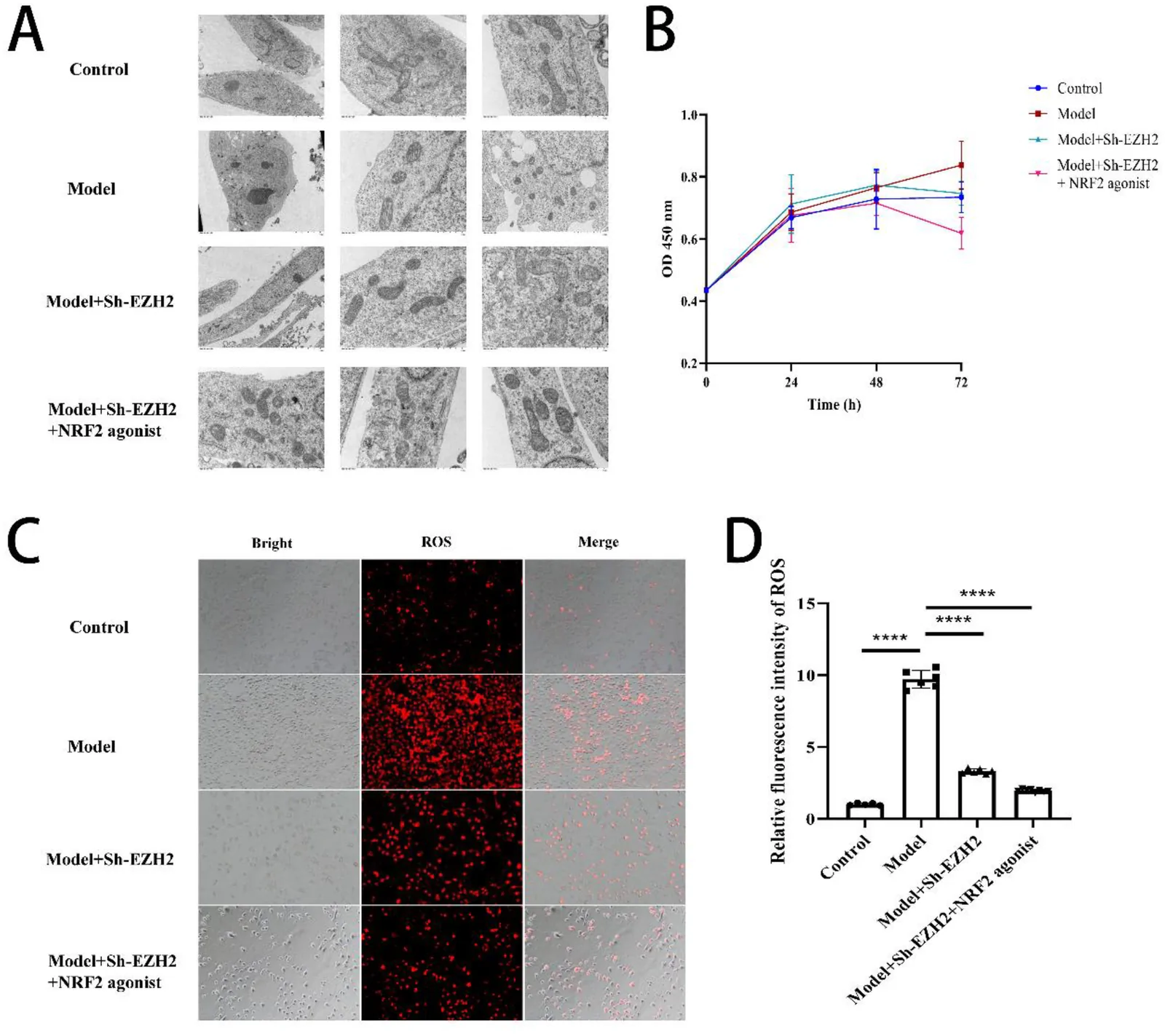
EZH2 knockdown attenuates intracellular oxidative stress and is accompanied by improved cell viability and mitochondrial morphology. **(A)** Representative transmission electron microscopy images of primary nasal polyp epithelial cells from the control, model, model plus Sh-EZH2, and model plus Sh-EZH2 plus NRF2 agonist groups. Cells were initially fixed, collected by gentle scraping and centrifugation, refixed, and subsequently processed for ultrathin sectioning and transmission electron microscopy. **(B)** Cell Counting Kit-8 assay showing relative cell viability at 0, 24, 48, and 72 h. **(C)** Representative DCFH-DA fluorescence images and **(D)** quantification of general intracellular reactive oxygen species. Data are presented as mean ± standard deviation. *P < 0.05, **P < 0.01, ***P < 0.001, and ****P < 0.0001 for the indicated comparisons.

### 3.5 AAV-m-EZH2-shRNA1 treatment is associated with changes in ferroptosis-related protein expression *in vivo*

Immunohistochemical analysis of nasal mucosa demonstrated that ACSL4, a key positive regulator of ferroptosis, was elevated in the model group, whereas GPX4, FTH1, and SLC7A11 protein levels were each significantly lower than in the control group (Figures 3A-D). Following administration of AAV-m-EZH2-shRNA1, GPX4, FTH1, and SLC7A11 were markedly upregulated, whereas ACSL4 was suppressed relative to the model group.

**Figure 3:**
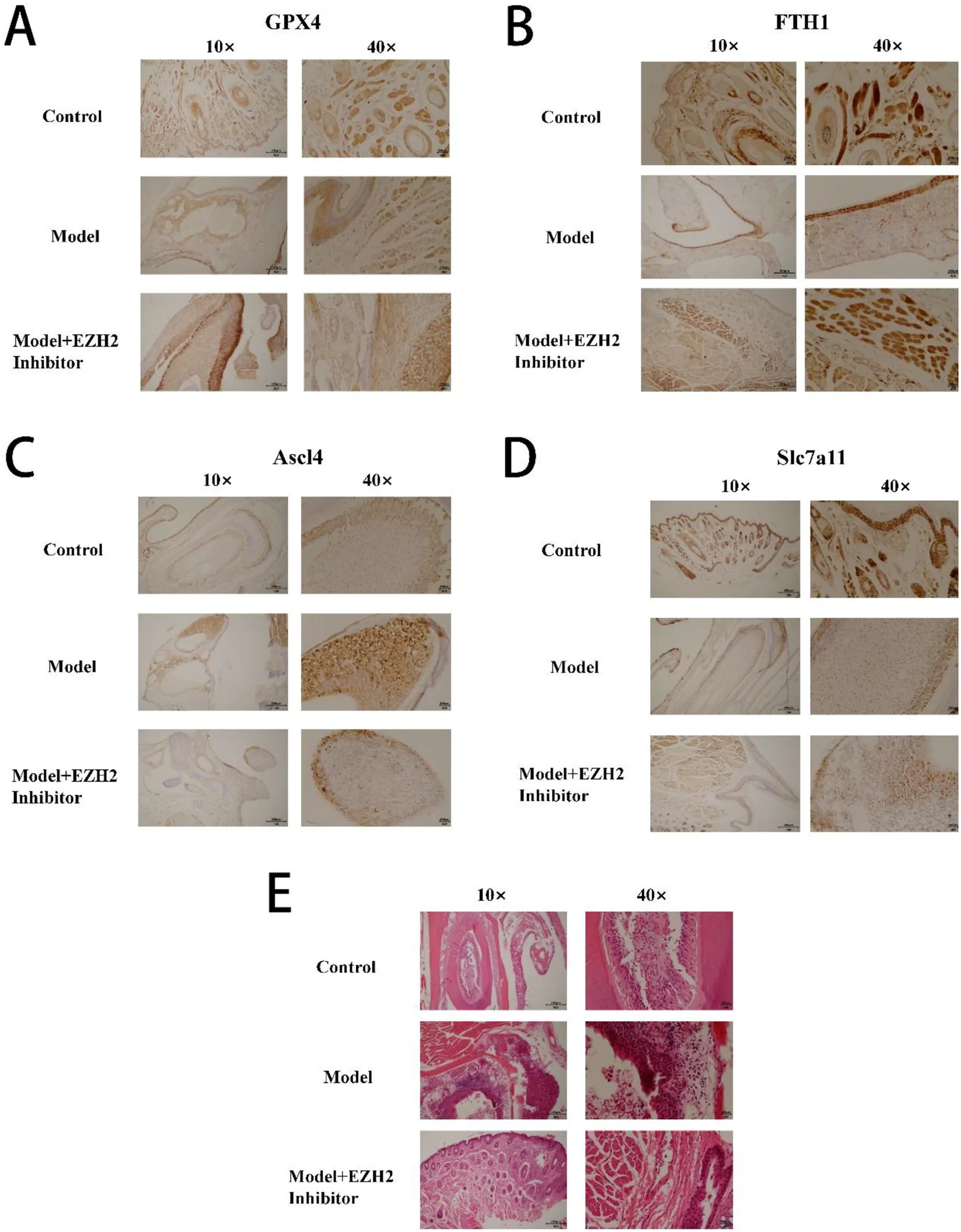
EZH2 knockdown modulates ferroptosis-associated protein expression and attenuates histopathological injury in eCRSwNP mice. **(A–D)** Representative immunohistochemical staining for GPX4, FTH1, ACSL4, and SLC7A11, respectively, at original magnifications of 10× and 40×. Brown diaminobenzidine staining indicates immunoreactivity, and nuclei were counterstained blue with hematoxylin. **(E)** Representative hematoxylin and eosin staining of nasal mucosa illustrating edema, eosinophil and lymphocyte infiltration, and polypoid hyperplasia. Data are presented as mean ± standard deviation. *P < 0.05, **P < 0.01, and ***P < 0.001.

### 3.6 EZH2 inhibition attenuates histopathological changes in eCRSwNP mice

Hematoxylin and eosin staining showed pronounced mucosal edema, inflammatory-cell infiltration, and polypoid epithelial changes in the model group. These histopathological abnormalities appeared less prominent in the AAV-m-EZH2-shRNA1-treated group (Figure 3E).

## 4 Discussion

This exploratory Brief Research Report identifies an association among elevated EZH2 expression, reduced NRF2-related antioxidant signaling, and ferroptosis-associated molecular and ultrastructural features in eCRSwNP. Across human tissues, primary cell experiments, and an ovalbumin/staphylococcal enterotoxin B-induced murine model, increased EZH2 expression coincided with altered antioxidant and ferroptosis-related markers, intracellular oxidative stress, mitochondrial injury, and sinonasal inflammation. EZH2 knockdown was accompanied by reversal of several of these changes. These convergent observations support the EZH2-NRF2 axis as a candidate regulator of oxidative epithelial injury.

The observation that EZH2 is elevated specifically in the eosinophilic endotype is consistent with evidence that EZH2 restrains type 2 immune responses. Loss of EZH2 in CD4-positive T cells has been shown to promote exaggerated eosinophilic airway inflammation and increased interleukin 4 and interleukin 5 production in murine models. EZH2 has also been reported to restrict natural killer T-cell pathogenicity relevant to spontaneous asthma-like disease, and reduced EZH2 expression in T helper cells has been associated with allergic rhinitis in humans (45, 46, 55). A separate line of evidence indicates that exosomal long non-coding RNA GAS5 can suppress EZH2 and favor T helper 2 polarization in allergic rhinitis, further supporting a T helper 2-restraining role for EZH2 under physiological conditions (56). The elevated EZH2 observed here in eNP tissue may therefore represent a compensatory but ultimately maladaptive epithelial and stromal response. Rather than protecting against type 2 inflammation, this response may affect oxidative epithelial injury through NRF2 suppression. This distinction likely reflects cell-type-specific and context-dependent EZH2 activity, as described in other inflammatory and fibrotic conditions, including inflammatory bowel disease, acute respiratory distress syndrome-associated pulmonary fibrosis, autoimmune hepatitis, and renal fibrosis, in which EZH2 inhibition consistently reduces tissue damage through mechanisms involving macrophage polarization and epithelial-mesenchymal transition (48, 49, 51, 57, 58). The broader relevance of EZH2 as a therapeutic target beyond oncology is reinforced by a recent comprehensive review describing its expanding role as a systemic disease modulator in fibrosis, autoimmunity, and neuroinflammation (48).

Co-immunoprecipitation detected EZH2 and NRF2 in the same protein complex. This finding supports biochemical co-association but does not establish direct binding or its functional consequences. Prior evidence that EZH2 can epigenetically silence NFE2L2, the gene encoding NRF2, and that reduced EZH2-mediated promoter methylation is linked to NRF2 overexpression in colon cancer suggests a context-dependent and potentially bidirectional relationship between these regulators (51). The polycomb repressive complex 2-independent, noncanonical capacity of EZH2 to engage transcription factors and other protein substrates, well documented in oncogenic settings, provides a plausible structural basis for an EZH2-NRF2 protein association in this study, in addition to any classical H3K27me3-dependent transcriptional repression of NFE2L2 (43, 44, 59). Pharmacological EZH2 inhibitors, including tazemetostat and valemetostat, are approved or in advanced clinical development for oncological indications, and EZH1 or EZH2 inhibition has recently been shown to enhance T-cell-mediated immunity in cancer models (45, 60–62). The pathway suggested here therefore raises the possibility of repurposing existing EZH2-targeting strategies for refractory eCRSwNP. However, the immunomodulatory consequences of systemic EZH2 inhibition for T helper 2 skewing would require careful evaluation, given the T helper 2-restraining function of EZH2 described above (45).

Downstream of NRF2, the marker pattern observed in this study aligns with established ferroptosis biology. GPX4 is the principal enzyme that detoxifies membrane phospholipid hydroperoxides, and its suppression is widely regarded as a central, rate-limiting event in ferroptosis execution. Its transcriptional induction by NRF2 has been documented across multiple tissue contexts (63–65). ACSL4 promotes the incorporation of long-chain polyunsaturated fatty acids into membrane phospholipids and thereby increases substrate availability for peroxidation; elevated ACSL4 together with suppressed GPX4 is considered a canonical ferroptosis signature, consistent with the reciprocal changes in these two markers observed in both the cell and animal experiments here (66, 67). SLC7A11 mediates cystine uptake required for glutathione synthesis, and its downregulation compromises the antioxidant capacity needed to counter lipid peroxidation. FTH1 sequesters labile iron and limits Fenton chemistry. Both are recognized transcriptional targets of the NRF2 antioxidant response element network, which may explain their coordinate suppression when NRF2 activity is diminished by EZH2 and their recovery upon EZH2 inhibition (68, 69). PTGS2, also known as cyclooxygenase 2, has been repeatedly validated as a biomarker that can increase in parallel with lipid peroxidation during ferroptosis across cancer and inflammatory contexts, consistent with its pronounced upregulation in eNP tissue and in the model group observed here (70, 71). The mitochondrial ultrastructural changes captured by transmission electron microscopy, including shrunken, electron-dense mitochondria with reduced cristae, further support a ferroptosis-associated morphological pattern but are not specific to ferroptosis (25, 26, 72).

The present findings also extend prior bioinformatic work that identified ferroptosis-related hub genes and regulatory RNA-binding proteins in chronic rhinosinusitis with nasal polyps by evaluating EZH2 loss of function in primary epithelial cells and a murine model (32–34). Within the broader context of ferroptosis biology in the airway, eosinophils themselves are susceptible to a related, noncanonical form of ferroptotic death that can be therapeutically exploited to relieve eosinophilic inflammation (27, 28, 73). This finding suggests that ferroptosis may exert cell-type-specific and even opposing effects on disease activity, promoting injury and barrier breakdown in epithelial cells but potentially promoting resolution of inflammation in eosinophils. This duality is consistent with the broader recognition that ferroptotic cell death can be either immunostimulatory or immunosuppressive depending on cellular context. It implies that therapeutic strategies targeting the EZH2-NRF2 ferroptosis axis in eCRSwNP should be designed to selectively protect epithelial integrity rather than uniformly suppressing ferroptosis across all cell populations (74–76). Additional airway evidence, including the observation that cold exposure aggravates allergic airway inflammation through epithelial ferroptosis and that eosinophil extracellular trap formation correlates with chronic rhinosinusitis severity, further situates the present findings within a coherent and expanding literature connecting oxidative epithelial injury, granulocyte activity, and type 2 airway disease severity (31, 77).

Two mechanistic interpretations of the observed EZH2/NRF2-associated pattern should be considered. In a canonical model, EZH2 could act through PRC2-dependent H3K27 trimethylation at the NFE2L2 promoter or other regulatory chromatin, leading to reduced NFE2L2 transcription (42–44). The observed changes in NFE2L2/NRF2 mRNA are compatible with this possibility but do not demonstrate it, because EZH2 chromatin occupancy and H3K27me3 enrichment at the NFE2L2 locus were not examined. Alternatively, EZH2 may participate in a noncanonical protein complex containing NRF2 and potentially influence NRF2 localization, stability, degradation, or transcriptional activity (42, 44, 59). The co-immunoprecipitation result is consistent with protein co-association but cannot establish direct binary binding, PRC2 independence, or a functional effect on NRF2. Thus, the current data cannot distinguish canonical H3K27me3-mediated regulation from a noncanonical association, and the two possibilities are not mutually exclusive. Chromatin immunoprecipitation or CUT&RUN for EZH2 and H3K27me3 at NFE2L2 regulatory regions, comparison of catalytic EZH2 inhibition with EZH2 depletion or catalytic-dead rescue, reciprocal co-immunoprecipitation, and direct NRF2 localization, stability, and activity assays will be required to resolve these alternatives.

The principal limitations of this study concern cell-death specificity and temporal directionality. First, the coordinated changes in GPX4, ACSL4, SLC7A11, FTH1, and PTGS2 overlap with molecular patterns frequently reported in ferroptosis. Similarly, mitochondrial shrinkage, increased membrane density, and reduced cristae have been described during ferroptosis. Nevertheless, neither these protein changes nor mitochondrial morphology is specific to ferroptotic cell death. Consistent with current recommendations for robust and reproducible ferroptosis research (23), functional rescue with Ferrostatin-1 or Liproxstatin-1, demonstration of iron dependence, and direct assessment of phospholipid peroxidation are required before ferroptosis can be established. Because these experiments were not performed, the present findings are described only as ferroptosis-associated features. DCFH-DA measures general intracellular oxidation and cannot substitute for direct phospholipid-peroxidation assays such as C11-BODIPY 581/591. Furthermore, MDA, 4-HNE, and the labile iron pool were not measured. Consequently, apoptosis, necroptosis, pyroptosis, and mixed regulated cell-death programs cannot be excluded. Second, EZH2, NRF2-related markers, and ROS were assessed under established cellular conditions without early time-course sampling. Therefore, the present study cannot determine whether ROS accumulation precedes EZH2 upregulation and NRF2 suppression, whether EZH2/NRF2 alterations initiate oxidative stress, or whether these events form a bidirectional feedback loop. NAC treatment was not performed, and the effect of ROS scavenging on EZH2 expression remains unknown. In addition, the use of an NRF2-activating compound after EZH2 knockdown cannot establish NRF2 dependence. Future studies should measure ROS, EZH2 mRNA and protein, total and nuclear NRF2, and downstream antioxidant effectors at 1, 3, 6, 12, and 24 h after a defined stimulus. These experiments should incorporate NAC, reciprocal ROS-induction approaches, and NRF2 loss-of-function in EZH2-silenced cells. The present study did not assess EZH2 or H3K27me3 occupancy at the NFE2L2 locus and therefore cannot determine whether the observed association reflects canonical PRC2-dependent repression, a noncanonical protein association, or both. Ferrostatin-1 or Liproxstatin-1 rescue, deferoxamine treatment, comparator cell-death inhibitors, C11-BODIPY 581/591-based phospholipid-peroxidation measurements, and assessment of the labile iron pool will also be required. The current observations should therefore be interpreted as preliminary and hypothesis-generating.

## 5 Conclusion

Elevated EZH2 expression was associated with reduced NRF2-related antioxidant signaling, increased intracellular oxidation, mitochondrial injury, and a ferroptosis-associated marker pattern in eCRSwNP. EZH2-targeting interventions were accompanied by changes in these measurements, but the present data do not establish ferroptotic cell death or a causal EZH2-NRF2-ferroptosis pathway. Functional rescue, demonstration of iron dependence, direct phospholipid-peroxidation measurements, and comparator cell-death experiments are required to determine the identity and mechanism of the observed cell-injury phenotype.

## Data Availability

The raw data supporting the conclusions of this article will be made available by the authors, without undue reservation.
