# Supplementary material for: Elevated EZH2 expression is associated with reduced NRF2-related antioxidant signaling and ferroptosis-associated features in eosinophilic chronic rhinosinusitis with nasal polyps
Source: Front Med (Lausanne). 2026 Oct 28;13:1966049. doi: 10.3389/fmed.2026.1966049 (PMC13751467; doi:10.3389/fmed.2026.1966049)
Supplement: Supplementary file 1 [file Supplementaryfile1.docx]

**Supplementary Figure S1**. EZH2 upregulation is associated with NRF2 suppression and a ferroptosis-associated marker profile in eCRSwNP clinical tissues. (A) Representative western blot images of EZH2, NRF2, GPX4, HMOX1, and PTGS2 protein expression in control, non-eosinophilic nasal polyp (NP), and eosinophilic nasal polyp (eNP) tissues. (B) Quantification of protein bands normalized to β actin (n=6 per group). (C) Quantitative reverse transcription polymerase chain reaction analysis of EZH2, NRF2, GPX4, HMOX1, and PTGS2 messenger RNA levels normalized to GAPDH (n=6 per group). Data are presented as mean ± standard deviation. *P<0.05, **P<0.01, ***P<0.001.
